# The McGill simulator for endoscopic sinus surgery (MSESS): a validation study

**DOI:** 10.1186/s40463-014-0040-8

**Published:** 2014-10-24

**Authors:** Rickul Varshney, Saul Frenkiel, Lily HP Nguyen, Meredith Young, Rolando Del Maestro, Anthony Zeitouni, Elias Saad, W Robert J Funnell, National Research Council Canada, Marc A Tewfik

**Affiliations:** Department of Otolaryngology, Head and Neck Surgery, McGill University, Montreal, Canada; Centre for Medical Education, McGill University, Montreal, Canada; Department of Medicine, McGill University, Montreal, Canada; Neurosurgical Simulation Research Center, McGill University, Montreal, Canada; Department of BioMedical Engineering, McGill University, Montreal, Canada; National Research Council Canada, Boucherville, Canada; Royal Victoria Hospital, 687 Ave Des Pins O., Rm E4.41, Montreal, Quebec H3A 1A1 Canada

**Keywords:** Rhinology, Endoscopic sinus surgery, Training, Education, Simulation, Virtual reality, Resident, Minimally invasive surgery, Haptic, Technical abilities, Performance metrics, Nasal model

## Abstract

**Background:**

Endoscopic sinus surgery (ESS) is a technically challenging procedure, associated with a significant risk of complications. Virtual reality simulation has demonstrated benefit in many disciplines as an important educational tool for surgical training. Within the field of rhinology, there is a lack of ESS simulators with appropriate validity evidence supporting their integration into residency education. The objectives of this study are to evaluate the acceptability, perceived realism and benefit of the McGill Simulator for Endoscopic Sinus Surgery (MSESS) among medical students, otolaryngology residents and faculty, and to present evidence supporting its ability to differentiate users based on their level of training through the performance metrics.

**Methods:**

10 medical students, 10 junior residents, 10 senior residents and 3 expert sinus surgeons performed anterior ethmoidectomies, posterior ethmoidectomies and wide sphenoidotomies on the MSESS. Performance metrics related to quality (e.g. percentage of tissue removed), efficiency (e.g. time, path length, bimanual dexterity, etc.) and safety (e.g. contact with no-go zones, maximum applied force, etc.) were calculated. All users completed a post-simulation questionnaire related to realism, usefulness and perceived benefits of training on the MSESS.

**Results:**

The MSESS was found to be realistic and useful for training surgical skills with scores of 7.97 ± 0.29 and 8.57 ± 0.69, respectively on a 10-point rating scale. Most students and residents (29/30) believed that it should be incorporated into their curriculum. There were significant differences between novice surgeons (10 medical students and 10 junior residents) and senior surgeons (10 senior residents and 3 sinus surgeons) in performance metrics related to quality (*p* < 0.05), efficiency (*p* < 0.01) and safety (*p* < 0.05).

**Conclusion:**

The MSESS demonstrated initial evidence supporting its use for residency education. This simulator may be a potential resource to help fill the void in endoscopic sinus surgery training.

## Introduction

Endoscopic sinus surgery (ESS) requires specialized technical skills involving complex spatial, perceptual and psychomotor performances [1]. Expertise in this minimally invasive surgery necessitates bimanual dexterity within a small 3-dimensional space [1], avoidance of key vital structures (i.e. orbits, brain and carotid artery), thorough applied knowledge of the intricate anatomy, and proficiency in maneuvering with the indirect visual aid of a 2-dimensional monitor [2]. Given the proximity of the paranasal sinuses to critical structures such as the orbits and skull base, it can be understood why ESS is the most frequent reason for otolaryngic surgical litigation in the United States [3], and why the rate of complications during ESS is higher in trainees when compared to attending physicians [4].

Those teaching ESS have found alternative modalities to the traditional apprenticeship training model such as cadaveric dissections and 3D silicone models [1]. However, the latter have substantial limitations with regards to the complex needs of ESS training, such as the lack of tissue mobility of rigid silicone models [5] and the inadequate representation of tissue strength in cadavers [6]. Virtual reality (VR) simulators solve these deficiencies, as well as offer a standardized environment for a trainee to repeat a procedure multiple times until proficiency is achieved [7]. Additional benefits of VR simulation documented in other surgical domains include the ability to objectively assess surgical skills without the need of a tutor [8], reduction of patient risk, and the standardization of residency training regardless of a particular institution’s practice profile or access to a cadaver laboratory [9]. VR simulation has been demonstrated to be beneficial in many surgical disciplines [2,10-12], including otolaryngology [13,14].

In the field of ESS, the first VR sinus surgery simulator, the ES3, was developed between 1995 and 1998 [15]. To date, rigorous published validation studies supporting use of ESS simulators in resident training derive uniquely from the ES3 [1,3]. However, it is no longer commercially available and there are only a few devices in existence [15]. Other simulators, such as the Dextroscope endoscopic sinus simulator [16] and the VOXEL-MAN [17], have yet to demonstrate evidence to support their use for training. Thus, there is an obvious need for a VR simulator with evidence of acceptability and validity to fill the void in ESS training.

The McGill Simulator for Endoscopic Sinus Surgery (MSESS) is a VR simulator that aims to address this issue. The objectives of this study were to assess the feasibility, usability, perceived value, and initial evidence supporting the validity of the simulator.

## Methods

### Description of the participants

Ethical approval was obtained from the Institutional Review Board at McGill University. Between May and October 2013, the following participants were recruited into the study: senior medical students (third or fourth year) and otolaryngology residents. The residents were divided into two groups: junior residents (PGY1-3 s) and senior residents (PGY4-5). The junior residents were grouped together as they had limited or no operative experience in ESS with less than 5 cases, whereas the senior residents had more than 5 cases. Furthermore, in order to have performance metrics from expert surgeons, 3 attending staff proficient in ESS (fellowship trained in rhinology or that perform an average of one day of ESS or skull base procedures every week) were also recruited.

Each user was given a brief tutorial concerning the functionality of the tools, as well as a video demonstrating the tasks to be performed and the danger zones within the nasal cavity. They were also given a 5-minute period to familiarize themselves with the movement and haptic feedback of the tools and the use of the pedals prior to beginning the simulated tasks.

### Description of the MSESS

The MSESS was created by the Department of Otolaryngology – Head and Neck Surgery at McGill University and the National Research Council of Canada. It was developed upon the NeuroTouch platform, which is a neurosurgery simulator made by the National Research Council of Canada [18,19]. Validity of the neurosurgery simulator as a training tool has previously been described [20]. The simulated 3D nasal model was rendered using a single patient’s CT scan. Each anatomic structure within the simulated 3D nasal model was coded separately as to allow specific measurements of performance at each point within the nasal cavity.

**Figure 1:**
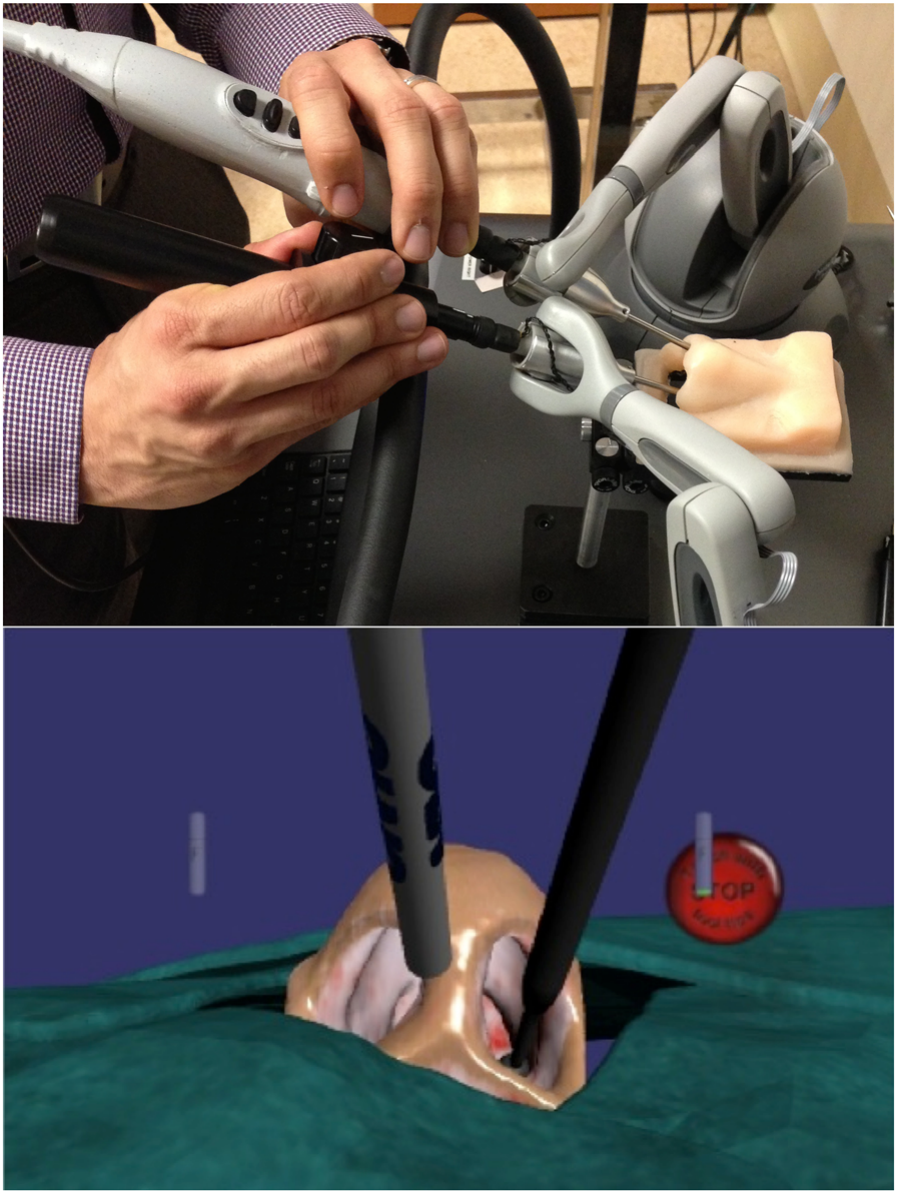
**Hardware of the MSESS.** View of the endoscope and the microdebrider handles (above) with VR view seen on the display monitor (below).

By providing a 0-degree endoscope in the non-dominant hand and a microdebrider in the dominant hand, the MSESS allowed the user to perform basic ESS tasks while viewing a virtual representation of the nasal cavity and the instrument tip on a flat panel display (Figure 1). A10-member panel of sinus surgeons and education experts opted to develop a microdebrider as the first simulated tool as it is commonly used in ESS, can perform a variety of tasks, and has a potential for serious complications [21]. The user received haptic feedback from the instruments, such as resistance from the contact of nasal tissues and vibration from the microdebrider activation.

A novel feature of the MSESS was its ability to simulate visual field blurring caused by soiling of the tip of the endoscope with nasal tissue contact. In this instance, the user had to activate an endonasal wash function via a foot pedal in order to regain clear visualization.

### Simulation tasks

**Figure 2:**
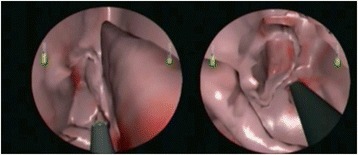
**VR representation of sinonasal cavity.** Views of an ethmoidectomy (left) and sphenoidotomy (right) using the microdebrider.

The tasks chosen to be evaluated on the MSESS included: 1) passing the endoscope from the nasal vestibule to the nasopharynx, 2) anterior ethmoidectomy, 3) posterior ethmoidectomy and 4) wide sphenoidotomy (Figure 2). The four tasks were chosen by the panel because they represented increasing levels of difficulty, and mimicked the step-wise approach found in sinus surgery where the surgeon typically addresses first the maxillary sinus, then the ethmoids, and finally the sphenoid sinus. The uncinectomy and maxillary antrostomy were not assessed since it cannot be safely performed with a microdebrider and other instruments have not yet been simulated.

### Performance metrics

Dimensions of quantitative data generated include constructs of *quality*, *efficiency*, and *safety.* Many of the metrics used to compare groups have previously been validated on the NeuroTouch platform [20]. A list of the metrics and their definitions can be found in Table 1.

**Table 1:** **Description of the performance metrics**

**Metric sphere**	**Definition**	**Metric**	**Units**
Quality	Completeness of targeted tissue removal	Amount of anterior ethmoids removed	Percentage (amount removed/total amount of relevant tissue)
		Amount of posterior ethmoids removed	Percentage
		Amount of sphenoid face removed	Percentage
Efficiency	Task performance with the least amount of unnecessary maneuvers	Time to complete tasks	Seconds
		Path length (endoscope)	Millimeters
		Path length (microdebrider)	Millimeters
		Fluctuation in distance between tips of endoscope & microdebrider (calculated by interquartile range)	Millimeters
		Frequency of microdebrider pedal activation	Number
		Amount of endonasal washes	Number
Safety	Amount of collateral damage	Amount of normal tissue removed, namely tissue over three critical “no-go” zones (lamina papyracea, skull base and optico-carotid recess)	Percentage
		Maximal force applied on skull base and lamina papyracea	Newtons

### Post-simulation questionnaire

After their simulation session, participants answered a questionnaire regarding their perceptions of simulator realism, potential educational benefits and skills practiced. Responses were collected via both a 10-point rating scale, anchored as appropriate for the question, and open-ended questions. Prior to implementation, this questionnaire had been sent to 5 faculty members on the research team to ensure that it was appropriate, intelligible, unambiguous, unbiased, complete, appropriately coded and aligned with our constructs of interest. Thereafter, a panel of 5 otolaryngologists and education experts assessed the questionnaire independently to validate it. Finally, residents and physicians were recruited to perform the initial pilot testing including assessment of intra-rater reliability for a final review of the post-simulation questionnaire.

### Data analysis

An average for each metric was calculated per group of participants (medical students, junior residents, senior residents, attending faculty), and used for comparison across participant groups.

Differences between groups’ performance metrics were first investigated using the analysis of variance - Kruskal Wallis Test. All metrics that showed a difference between groups were then sub-analyzed using the Mann-Whitney test to demonstrate which groups showed a difference (*p* < 0.05 was considered significant). Descriptive statistics were used to analyze the quantitative portion of the questionnaire, while content analysis and thematic description was applied to qualitative data.

## Results

### Participants

10 medical students, 10 junior residents, 10 senior residents and 3 attending staff agreed to participate in the study. All the participants completed the required simulation tasks, as well as the post-simulation questionnaire.

### Post-simulation questionnaire

Data relating to the assessment of perceived realism and educational value of the MSESS are presented in Tables 2 and 3, respectively. Participants across all groups, on average, rated items related to the realism of the MSESS at least 7 on a 10 point-rating scale, corresponding to the anchor “realistic” (Mean =7.97 ± 0.29). Similarly, participants across all groups rated items related to the perceived educational value of the MSESS at least 7 on a 10, corresponding to “useful” (Mean =8.57 ± 0.69).

**Table 2:** **Perceived assessment of the realism of the MSESS**

		**Medical students**	**Junior residents**	**Senior residents**	**Attending faculty**
		**Mean score (SD)**	**Mean score (SD)**	**Mean score (SD)**	**Mean score (SD)**
**Appearance of VR nasal model**	Nasal cavity	8.0 (0.67)	7.6 (1.83)	8.11 (1.53)	7.67 (0.57)
	Sinuses	7.9 (0.73)	7.7 (1.83)	8.11 (1.45)	7.67 (0.57)
	Medialization of turbinate	8.3 (0.82)	7.3 (1.88)	8.0 (1.58)	7.33 (0.57)
**Appearance and functionality of tools**	Microdebrider	8.4 (0.84)	7.7 (1.63)	8.33 (1.11)	7.33 (0.57)
	Suction on microdebrider	7.7 (0.82)	7.6 (1.83)	8.11 (1.26)	8.33 (0.57)
	Physical tool handles	8.5 (1.18)	7.4 (1.83)	7.89 (1.45)	8.33 (0.57)
	Haptic feedback	7.2 (1.22)	7.8 (1.75)	7.89 (1.16)	7.67(0.57)
	Endonasal wash	8.6 (1.07)	7.3 (1.57)	8.11 (0.93)	8.66 (0.57)
**Ability to simulate surgical steps**	Anterior ethmoidectomy	8.5 (0.53)	7.9 (1.63)	8.22 (0.83)	8.33 (0.57)
	Posterior ethmoidectomy	8.5 (0.53)	7.7 (1.63)	8.22 (0.97)	8.0 (0)
	Sphenoidotomy	8.5 (0.71)	7.5 (1.84)	8.22 (0.97)	8.33 (0.57)

Scores were on a 10-point rating scale. The anchors to the scale were 1 = No resemblance at all, 4 = Some resemblance, 7 = Realistic, 10 = Real-Life.

**Table 3:** **Perceived educational value of the MSESS**

		**Medical students**	**Junior residents**	**Senior residents**	**Attending faculty**
		**Mean score (SD)**	**Mean score (SD)**	**Mean score (SD)**	**Mean score (SD)**
**Learn theory**	Anatomy	9.4 (0.84)	8.3 (2.0)	9.0 (1.0)	8.67 (0.57)
	Steps - anterior ethmoidectomy	9.7 (0.67)	8.2 (1.93)	8.78 (1.09)	7.67 (0.57)
	Steps - posterior ethmoidectomy	9.7 (0.67)	8.4 (1.89)	8.78 (1.09)	7.66 (1.15)
	Steps - sphenoidotomy	9.6 (0.69)	8.2 (1.75)	8.45 (1.23)	7.0 (1.0)
**Practice technical skills**	Hand-eye coordination	9.5 (0.84)	8.1 (2.18)	9.0 (1.32)	7.67 (1.41)
	Bimanual dexterity	9.5 (0.84)	8.1 (2.28)	8.89 (1.36)	8.0 (0)
	Efficiency	9.6 (0.69)	7.9 (1.75)	8.44 (1.23)	7.33 (2.08)
**Safety**	Identify	9.4 (0.84)	8.7 (1.94)	8.0 (1.64)	9.0 (1.0)
	No-go zones^1^				

Scores were on a 10-point rating scale. The anchors to the scale were 1 = Not at all useful, 3 = Minimally useful, 5 = Adequate, 7 = Useful, 10 = Extremely useful.

^1^No-go zones referred to the lamina papyracea, orbital fat, skull base and optico-carotid recess.

All medical students (*n* = 10/10) felt that the MSESS would be useful for their level of training, as compared to 80% of junior residents (*n* = 8/10) and 80% (*n* = 8/10) of senior residents. Similarly, 100% of medical students (*n* = 10/10) stated that the MSESS would be a useful adjunct to their surgical curriculum, as did 80% of junior residents (*n* = 8/10) and 80% of senior residents (*n* = 8/10). Finally, when asked if the MSESS should be readily available for their rhinology surgical education, 29/30 students and residents responded yes.

The responses to open-ended questions for strengths of the simulator were grouped into three main themes: the realism of the VR model, the ability to practice bimanual technical skills and the necessity for such simulators to complement traditional teaching modalities. Weaknesses related to perceived imprecision of fine tool movements and the lack of bleeding in the VR model.

### Performance metrics

#### Quality

**Figure 3:**
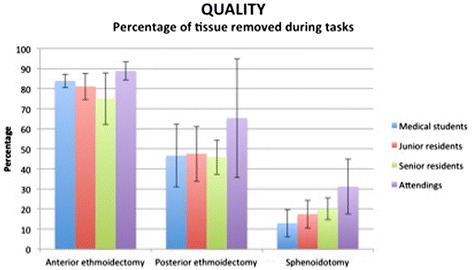
**Percentage of tissue removed during simulation tasks.** The graph represents means +/- SD. There was no statistically significant difference (*p* > 0.05) between all 4 groups for all three surgical tasks. When combining the groups into novices (students and junior residents) and senior surgeons (senior residents and attending faculty), there was a statistically significant difference for the wide sphenoidotomy (*p* = 0.01).

There was no statistically significant difference (Figure 3) between all 4 groups with respect to the surgical completeness of the anterior ethmoidectomy, posterior ethmoidectomy and wide sphenoidotomy (*p* > 0.05). However, when combining the groups into novices (medical students and junior residents) and senior surgeons (senior residents and attending faculty), there was a significant tendency towards making a wider sphenoidotomy with increasing level of expertise (*p* = 0.01).

#### Efficiency

**Figure 4:**
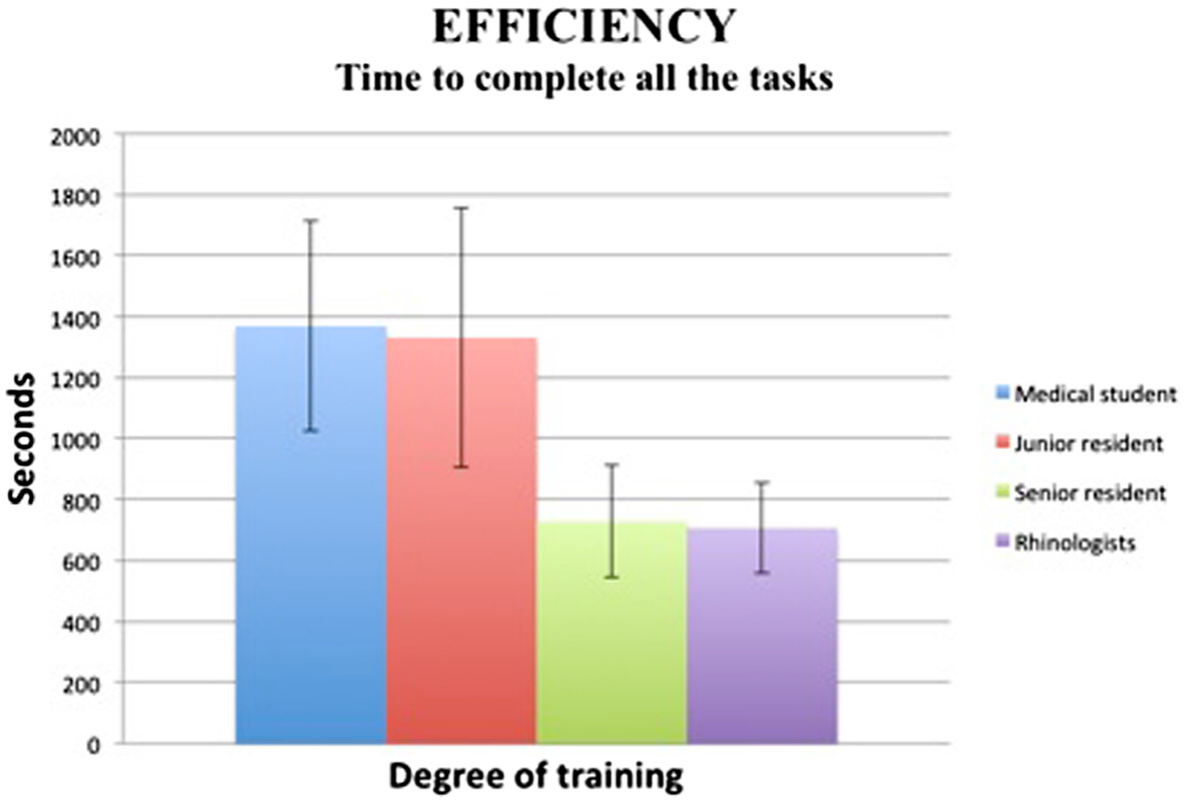
**Time to complete the simulation tasks.** The graph represents means +/- SD. Statistically significant difference (*p* < 0.005) between junior residents and senior residents. No difference between medical students and junior residents, nor between senior residents and attending faculty.

**Figure 5:**
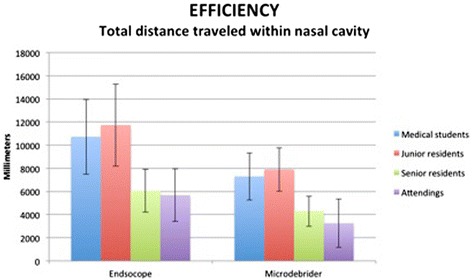
**Path length (Distance travelled within nasal cavity).** The graph represents means +/- SD. Statistically significant difference between junior residents and senior resident for both the endoscope (*p* < 0.001) and the microdebrider (*p* < 0.001). No difference between medical students and junior residents, nor between senior residents and attending faculty.

Time required to complete the tasks is presented in Figure 4. The only significant difference was between the junior residents group and the senior residents (*p* < 0.005). With regards to path lengths for the endoscope and the microdebrider (Figure 5), both metrics demonstrated a statistically significant difference between junior residents and senior residents (*p* < 0.001).

**Figure 6:**
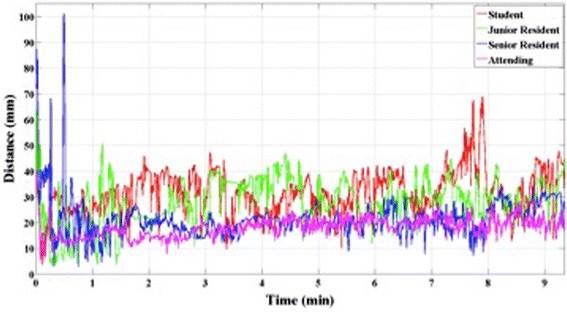
**Distance between tool tips through the simulation tasks.** The senior residents and attending faculty demonstrate far less fluctuation than medical students and junior residents.

The average fluctuation in distance between the tips of the endoscope and the microdebrider for the medical students, junior residents, senior residents and attending faculty were 12.64 ± 3.04 mm, 12.23 ± 3.91 mm, 9.91 ± 2.45 mm and 6.98 ± 2.39 mm, respectively. There was a statistically significant difference between junior residents and senior residents (*p* < 0.01). A graphical illustration of distance between tool tips for users of different levels of expertise is presented in Figure 6.

The frequencies of activation of the microdebrider pedal for medical students, junior residents, senior residents and attending faculty were 188 ± 65, 173 ± 64, 87 ± 37 and 104 ± 17 times, respectively. There was a significant difference between junior residents and senior residents (*p* < 0.001). With regards to the frequency of use of the endonasal wash, there was a tendency towards less use with increased training: 17 ± 12, 12 ± 10, 7 ± 3 and 2 ± 2 times, respectively. Again, there was a statistically significant difference between junior residents and senior residents (*p* < 0.01) for these metrics.

All the metrics related to efficiency showed a difference between junior residents and senior residents. However, there were no significant differences between medical students and junior residents, nor between senior residents and attending faculty.

#### Safety

**Figure 7:**
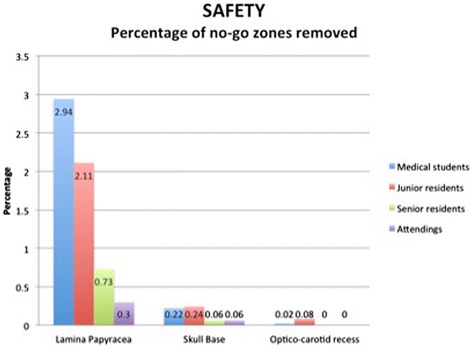
**Percentage of no-go zones removed.** The graph represents means. Statistically significant difference between junior residents and senior residents for the percentage of lamina papyracea removed (*p* < 0.005). No difference between medical students and junior residents, nor between senior residents and attending faculty. No statistical difference for other no-go zones.

With regards to violation of the no-go zones (Figure 7), there was a significant difference between junior residents and senior residents with regards to the percentage of lamina papyracea mucosa removed (*p* < 0.005). With respect to the skull base, all four groups removed a minute amount of tissue (<0.25%), with no significant difference (*p* > 0.05). Medical students and junior residents removed 0.02% and 0.08% of the mucosa surrounding the optico-carotid recess, whereas seniors and attending faculty had no contact with that region.

Medical students and junior residents applied a maximal force of 0.75 ± 0.67 N and 0.15 ± 0.31 N on the lamina papyracea, respectively. The senior residents and attending faculty applied a negligible force on the lamina. The maximal force applied on the skull base was 0.93 ± 0.54 N, 0.53 ± 0.68 N, 0.24 ± 0.49 N and 0 N, respectively, with increasing level of training. The only significant differences were between junior residents and senior residents (*p* < 0.05).

## Discussion

Attributes available on the MSESS include increasing task difficulties, blurring of the camera field with tissue contact, an endonasal wash function, a microdebrider, and mobility of the nasal tissues. Compared to previous sinus simulators, we believe that a combination of these attributes allow the user to experience a more realistic, higher fidelity physical and visual environment. Furthermore, measurement of performance metrics from both hands independently, including measures of bimanual dexterity, as well as the ability to identify contact with danger zones allow a more elaborate performance assessment.

Given the lack of available ESS simulators with enough data supporting validity as a training tool, the current initial validation study of the MSESS is the first step towards filling this void. In fact, we demonstrated that participants from all levels of training found the simulator to be realistic in terms of visual appearance and content. They also responded that the simulator allowed them to practice the technical skills required for ESS. Furthermore, through analysis of the performance metrics, not unexpectedly, novices fared significantly worse than senior surgeons in measures of operative efficiency, which echoes previous reports in studies of surgical simulators [22,23]. Similarly, within the field of ESS simulation, Edmond showed that novice surgeons without ESS experience performed worse on simulation training [24].

The inability of the performance metrics to differentiate medical students from junior residents is likely related to the fact that residents do not routinely perform ESS until their senior years. Moreover, the lack of difference between senior residents and attending faculty on the performance metrics may be related to the small number of attending faculty (n = 3), as some metrics, namely those related to efficiency, demonstrated a tendency towards improved performance by the attending faculty compared to senior residents. Nevertheless, these findings may indicate that the learning curve for performing simple ESS tasks is relatively steep and that the MSESS may be most valuable for junior residents prior to direct patient contact.

Research has demonstrated that recognition of anatomy with an endoscopic view is one of the more challenging parts of ESS [25]. In fact, authors have reported that a strong familiarity with intranasal 3D relationships and spatial boundaries are more vital for operative success than the technical skills of sinus surgery [1,24] Thus, one of the main focuses during the development of the MSESS was to develop a simulated nasal model that was as realistic as possible, reflected by the participants’ high assessment scores on the questionnaire.

Furthermore, the MSESS was tailored to help train users on complex technical skills, such as bimanual dexterity and hand-eye coordination, which are prerequisite skills for ESS [2]. The fact that there was decreasing fluctuation in the distance between the two tool tips with increasing degree of experience suggests that there is a notable learning curve for bimanual dexterity, which has previously been shown to vary with level of expertise [26]. In fact, Narazaki et al. demonstrated that experts outperformed novices in terms of bimanual dexterity skills significantly on a laparoscopic surgery simulator and advocated for its’ testing as a means to objectively assess the proficiency of a surgeon [27].

In order to demonstrate validity as an educational tool, many studies on simulators have aimed to show a difference between users of different degrees of experience [28]. The latter shows that the simulator actually measures the technical skills that are intended to be measured [2]. Previous simulators have demonstrated this metric in support of “construct validity”, including simulators for surgical skills in laparoscopic surgery [29], bone sawing skills [30], neurosurgery [20] and ESS [9,31], as well as diagnostic skills such as coronary angiography [32], obstetrical ultrasonography [33] and colonoscopy [34].

The performance metrics recorded by the MSESS – divided into measures of quality, efficiency and safety – allowed us to test this form of validity. With regards to quality, users across all groups removed similar percentages of the anterior and posterior ethmoids. This is not surprising as removing tissue is not a difficult task in and of itself, but doing so efficiently and safely differentiates a novice from an experienced surgeon. Furthermore, a notable tendency was observed towards increasing extent of the sphenoidotomy with advancing level of expertise, most likely explained by the fact that more experienced surgeons had a heightened awareness of what is safe to remove in the sphenoid sinus and what are danger zones for injury to critical structures such as the optic nerve and carotid artery. In contrast, junior surgeons are more apprehensive in this region and thus elect to be more conservative.

Moreover, despite this suspected apprehensiveness demonstrated by juniors, users in the medical students and junior residents groups made contact with “no-go” zones such as the lamina papyracea and optico-carotid recess more commonly. Edmond demonstrated that the most discriminating performance factor during the novice mode on a previous ESS simulator was the ability to avoid hazards [24], which is a skill that senior surgeons learn with experience and thorough anatomy knowledge. Through recognition of these errors, novice surgeons may learn to avoid trauma to collateral tissue. In fact, decreased tissue injury during technical skills assessments after training on VR simulators has previously been demonstrated [7].

Endoscopic sinus surgeons are cognizant of the fact that the lamina papyracea and skull base are sensitive areas due to their fragility as well as the structures that they protect, thus it is important to be able to measure the amount of force that is applied upon them by our tools. Our study demonstrated that there was a significant difference in the maximal force applied between novice surgeons and more senior surgeons. The importance of force measurements also highlights a pitfall of training on cadaveric tissues, which do not adequately estimate the force necessary to perform endoscopic sinus procedures [6] and thus, do not show trainees the acceptable force allowed during ESS. Although novice surgeons applied more force in our study, the next step would be to determine the critical amount of force that would be needed to cause damage and assess whether the increased force applied by junior surgeons is truly clinically dangerous.

The benefits of simulation training are highlighted by the difference in efficiency between junior and senior surgeons. Simulation training allows residents to be more efficient, thus saving time in the operating room, where time is limited and expensive [35]. The premise of training on the MSESS is that if a junior can practice ESS on the simulator, he begins hands-on training at an earlier stage, prior to direct patient contact [24] and thus is better prepared when in the operating room. Furthermore, with decreased resident working hours [36], it is even more essential to have alternative methods for junior surgeons to practice their technical skills.

## Conclusion

The MSESS demonstrated initial evidence supporting its use for residency education with regards to being a realistic and useful training tool. The performance metrics relating to quality, efficiency and safety also demonstrated a dichotomy between novice and senior surgeons. The next step in this validation process will be to compare the MSESS to other teaching modalities, including cadaveric dissection which is currently the gold standard of ESS training; to assess the predictive validity of the MSESS; and to demonstrate translation of technical skills in the setting of live patient interactions.
